# 3D printing-guided chest wall reconstruction – a case report and literature review

**DOI:** 10.1016/j.jpra.2026.03.019

**Published:** 2026-03-19

**Authors:** Amy Yoon, Danial Bahudin, Wingchi Lo, Michael Wagels

**Affiliations:** a Division of Plastic Surgery, Princess Alexandra Hospital, 199 Ipswich Road, Woolloongabba, QLD 4102, Australia; b Australian Centre for Complex Integrated Surgical Solutions (ACCISS), Princess Alexandra Hospital, 199 Ipswich Road, Woolloongabba, QLD 4102, Australia; c Division of Cardiothoracic Surgery, Princess Alexandra Hospital, 199 Ipswich Road, Woolloongabba, QLD 4102, Australia; d The University of Queensland, School of Medicine, Herston, QLD 4006, Australia

**Keywords:** Chest wall reconstruction, 3D-printing, Surgical guide, Case report, Patient-matched medical device

## Abstract

Large chest wall defects may be challenging to reconstruct and requires consideration of skeletal stabilization. Whilst use of synthetic materials has been described, the risks of infection and implant failure resulting in removal are omnipresent. The vascularized fibula free flap is an autologous alternative. We present the first reported application of 3D-printed patient-matched fibula marking guide to facilitate reconstruction of a large sternal defect with an autologous vascularized fibula free flap.

A 56-year-old patient developed paradoxical chest wall movements with chest pain following a partial sternectomy for sternal fibrous dysplasia. The primary defect was reconstructed with a synthetic implant, complicated by infection requiring hardware removal. Cross-sectional imaging confirmed herniation of lung contents through the defect. Virtual surgical planning was undertaken to define the best configuration for available autologous bone options. A single free fibula twice osteotomized and configured into a triangle with 3 segments was chosen to close the defect. A patient-matched fibula marking guide was designed and 3D-printed for intra-operative use, and titanium plates were pre-contoured using a life-sized 3D-printed chest wall model. Surgery proceeded without complications, with reported resolution of symptoms post-operatively.

Current literature on 3D printing technology for chest wall reconstruction primarily focuses on synthetic implants rather than autologous tissue transfer. Whilst autologous bone grafting for sternal defects is well described, only three cases have reported the use of vascularized fibular free flap reconstruction.

This case demonstrates the feasibility of integrating a 3D-printing workflow in the surgical planning of autologous vascularized sternal reconstruction.

## Introduction

Large anterior chest wall defects, often secondary to oncologic resection, can compromise skeletal and respiratory function.^1^^,^^2^ Reconstruction aims to restore skeletal stability, soft tissue coverage, respiratory mechanics, and protection of intrathoracic structures.^1^^,^^2^ This is important for defects greater than four consecutive ribs or 5 cm to prevent paradoxical breathing.^3^

Whilst synthetic materials are popular for their ease of use, autologous bone reconstruction provides superior bio-integration and reduced infection profile.^4^ However, manual intra-operative bony contouring can be challenging, imprecise and iterative, prolonging operative and ischemic time.^5^^,^^6^ For larger defects, it can be unclear whether one donor site will provide sufficient length.

Three-dimensional (3D) printing technology addresses this limitation through pre-planned osteotomies, reducing intra-operative trial-and-error.^5^, ^6^, ^7^ However, its application in autologous sternal reconstruction is limited. We present a case employing virtual surgical planning (VSP) and 3D-printed surgical guides to reconstruct a large, complex sternal defect with lung herniation, with the aim to provide a reproducible template for similar cases. This case report is compliant with the CARE guidelines.^8^

## Case presentation

A 56-year-old male was referred for increasing sternal pain after a fall. Seven months prior, he had a complex partial sternectomy for sternal fibrous dysplasia. A wide margin was taken, sacrificing the internal mammary arteries (IMA), and the defect was reconstructed with a double layer prolene mesh, methylmetacrylate and pectoralis major muscle advancement flaps. However, this was complicated by surgical site infection, requiring removal of the sternal reconstruction.

**Figure 1:**
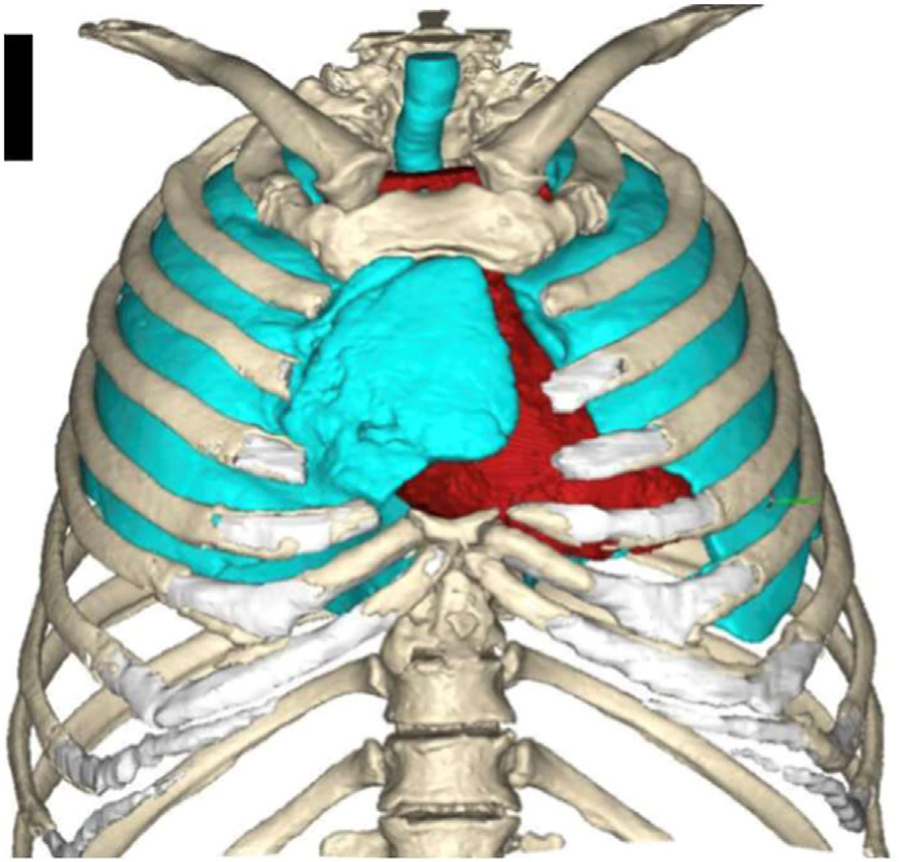
3D models of the sternal defect on Materialize 3-Matic. Model with soft tissue mask removed to show bony anatomy and mediastinal structures including herniated lung.

The patient had type-2 diabetes mellitus, iron deficiency anemia, and hypothyroidism, and was a former smoker. At the time of his fall, a chest CT demonstrated no acute findings. However, since then, he reported worsening sternal pain and new paradoxical chest wall movements on coughing, limiting his daily activities. On examination, the cutaneous wound had completely healed but there was a bulge over the sternal region, more pronounced with the Valsalva manoeuvre. This time, chest CT demonstrated herniation of the right middle lobe through the sternal defect (Figure 1). After discussions at a multidisciplinary team meeting, the decision was for sternal reconstruction via fibula free flap.

**Figure 2:**
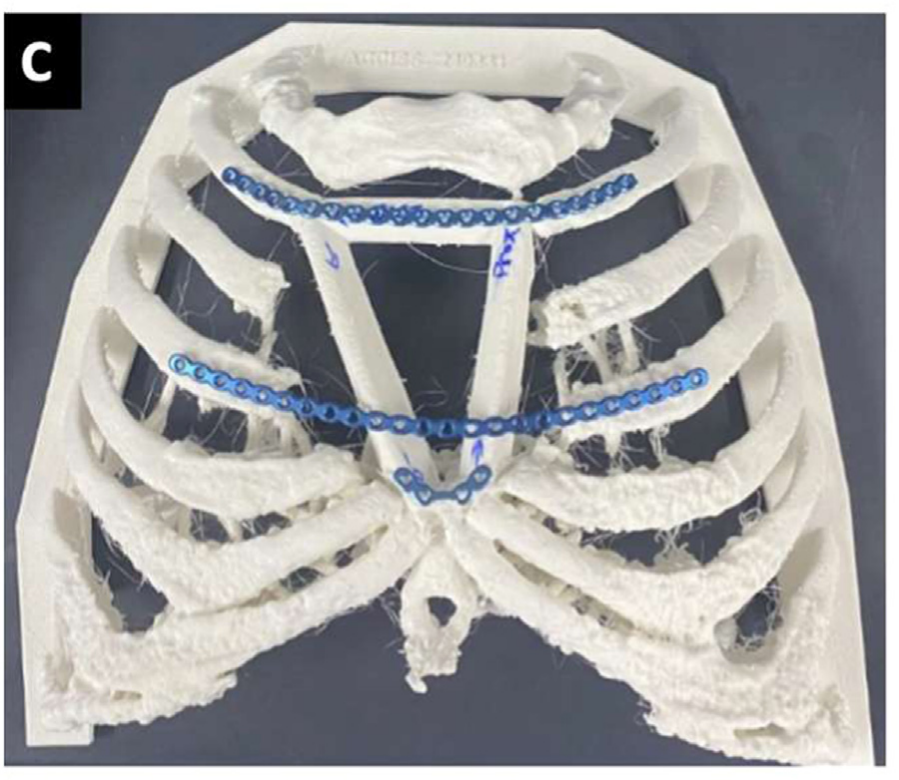
Pre-contouring of titanium plates. A 3D-printed life-sized model of the chest wall with the fibula segments superimposed onto the sternal defect. Titanium plates are contoured using the 3D printed model prior to the surgery. The final pre-contoured plates prior to sterilization for surgery.

The defect’s 3D geometry made it challenging to define fixation points for the plating system using conventional 2D-medical imaging methods. The hospital’s point of care team was consulted for VSP and their medical device manufacturing service. Multiple osteotomy configurations were iteratively tested to determine optimal defect coverage and bone-to-bone contact. The final decision was for a triangular configuration with three fibula segments, specifically angled to match the contour of the sternal defect and optimize plate fixation, permitting motion at a sterno-neomanubrial pseudoarthrosis (Figure 2). A marking guide with four marking locations was designed–the proximal border, middle notch, distal border, and distal pointer tips, each corresponding to planned osteotomy sites. Pointers were placed on each end to ensure correct guide placement. The middle notch had a wedge design for precise wedge osteotomy. The guide’s final design was printed using FormLab’s BioMed Clear Resin material. A full-scale anatomical model of the chest wall with the finalized osteotomy plan was also 3D-printed using polylactic acid (PLA) material for pre-operative plate bending and surgical rehearsal (Figure 2). Both the resin marking guide and the pre-contoured fixation plates were sterilized for surgery.

A midline sternotomy incision was made through pre-existing scar. Pectoralis major advancement flaps were separated and reflected. The herniated right lung was dissected off the pectoralis major and pericardium and reduced into the thoracic cavity. The hernial orifice was covered with prolene mesh. Thymic target vessels were prepared for microvascular anastomosis (MVA) as both IMAs were previously ligated. The flap was raised with a cuff of flexor hallucis longus. A skin paddle was not required. Proximal and distal cuts on the fibula were marked using the sterilized marking guide. Remaining markings and osteotomies were performed on a back table. The triangular construct was wired with 26 g pre-stretch dental-wire before transferring the flap to the chest for MVA.

**Figure 3:**
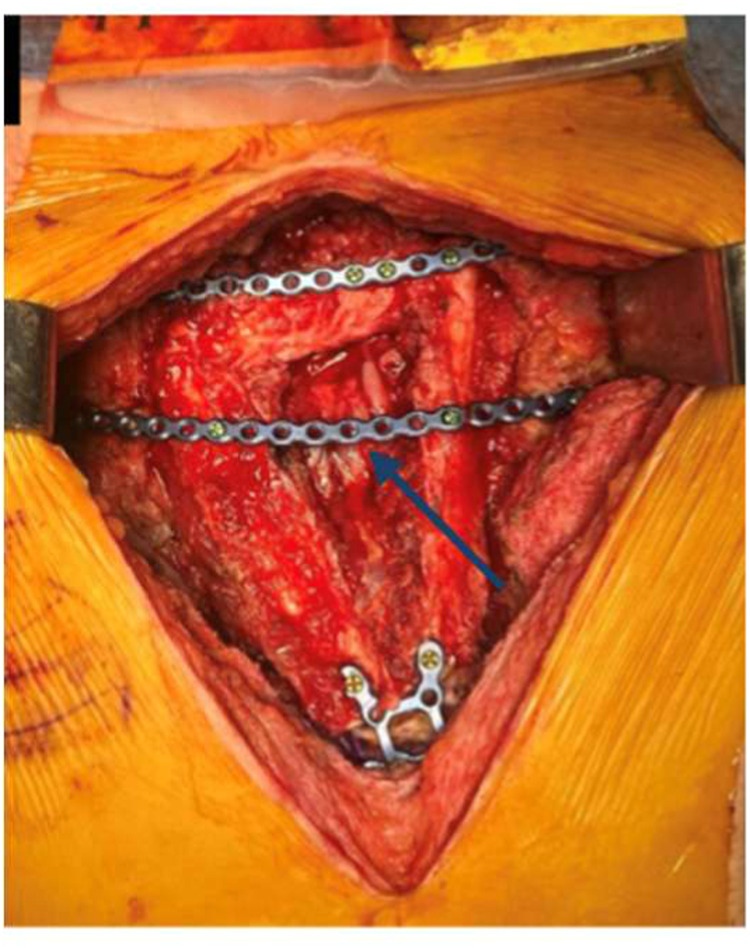
Intra-operative clinical image. The fixation of the vascularized fibula free flap onto the sternal defect using pre-bent titanium plates. Blue arrow to additional plate used to prevent anterior prolapse of long segments of triangle at superior margin of defect.

A window was cut in the prolene mesh to facilitate MVA. Anastomosis was challenging due to motion from underlying mediastinal structures. Flap pedicle length was insufficient for primary anastomosis, necessitating vein graft. Arterial anastomosis was performed end-to-end peroneal artery to thymic artery via vein graft from the dorsum of the foot. Venous anastomosis was configured similarly. The pre-bent plates were screwed in to secure the flap to the chest wall. An additional plate was added to prevent anterior prolapse of the long segments of the triangle at the superior margin of the defect (Figure 3). Pec major flaps were re-approximated over the plated fibula segments and cutaneous wound closed.

**Figure 4:**
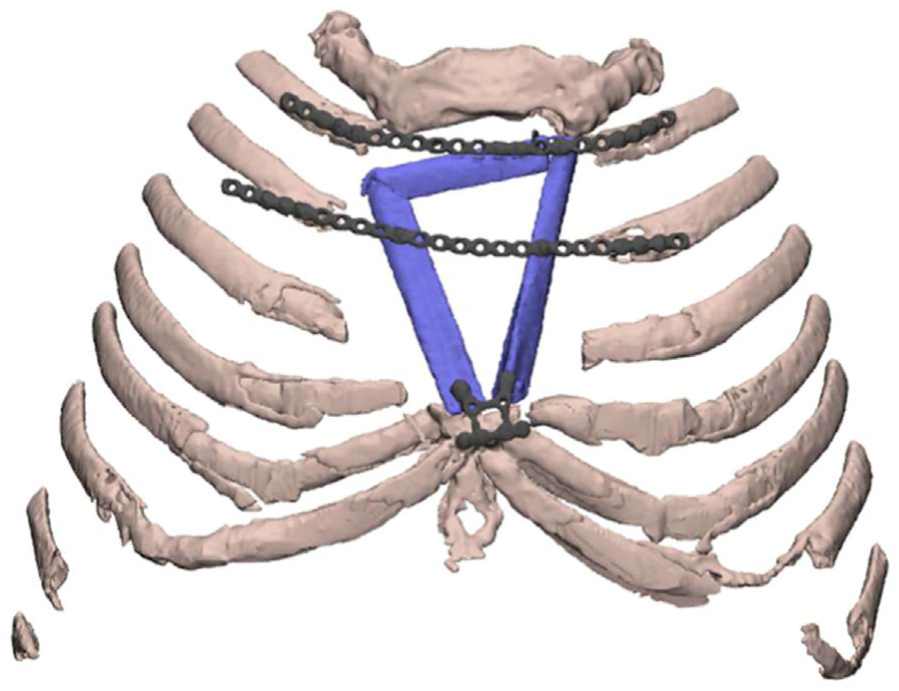
Post-operative imaging of the reconstructed chest wall. Virtual model of the chest wall using post-operative CT showing failure of right superior corner to meet 2nd rib and proud plate over lateral part of 3rd rib. This was the plate that had not been bent prior to surgery.

He remained in hospital for 37-days prior to discharge. Post-operative imaging confirmed accurate flap positioning, satisfactory positioning of fixation and no evidence of lung herniation (Figure 4). At 3-weeks post-operatively, he reported resolution of presenting symptoms. At 5-months, he remained stable with no stigmata of infection.

## Discussion

Large sternal defects following oncologic resection or sternal dehiscence remain a surgical challenge. Reconstruction requires both soft tissue coverage and bony stabilization in all vectors of chest wall motion, especially in the vertical ‘bucket-handle’ movement of lateral ribs and movement of the sternum in the anteroposterior plane. Current 3D-printing applications in this area predominantly focus on synthetic implants, leaving a gap in literature on autologous approaches.

Pectoralis major and rectus abdominis flaps are commonly used for reconstruction due to reliable blood supply, proximity and ease of harvest. However, myocutaneous reconstruction without skeletal stabilization may be inadequate, risking paradoxical movement, respiratory compromise, chronic pain, and herniation of mediastinal viscera with minor trauma. Although 3D-printed titanium implants allow for personalized approaches to skeletal restoration, they carry risks of foreign body complications, potentially requiring removal, and may not support normal pulmonary mechanics. Autologous vascularized bone is a potential solution via tissue integration and remodeling in response to loading. Currently, only three case reports describe vascularized fibula free flap reconstruction for sternal defects–two using double-barrel techniques,^3^^,^^9^ and one with triple-barrel approach.^10^ All reported favorable outcomes, but none utilized VSP or 3D-printing.

Our case presents application of VSP and a 3D-printed patient-matched guide for autologous vascularized sternal reconstruction. The guide allowed optimal use of a single fibula for reconstruction, providing pre-operative certainty, reducing intra-operative trial-and-error, and minimizing donor site morbidity. The configuration of fibula segments was biomechanically purposeful. The transverse component spanning across bilateral 2nd ribs re-established horizontal continuity. The V-shaped segments restored vertical rigidity to the heads of the lower ribs. These facilitated bucket-handle movements of ribs 3–8, permitting AP expansion at the sternomanubrial junction. Post-operatively, the patient demonstrated a stable chest wall and resolution of symptoms. He also found the 3D-printing technology helpful in understanding his defect and planned surgery, highlighting its potential as an adjunct to informed consent.

As a single-case study, generalizability is limited and outcomes may vary depending on defect size and surgical expertise. Furthermore, the execution was not entirely perfect as an unplanned plate to the 3rd rib was required due to short right posterolateral fixation point, underscoring that intra-operative adaptability remains essential despite detailed planning. Long term follow-up is necessary to access extent of bony union, and cost of VSP and 3D-printing represent widespread adoption barriers. Further prospective studies comparing 3D-printed guided and freehand approaches are required to validate this technique in complex sternal reconstruction.

## Conclusion

In conclusion, we present a novel application of a 3D-printed patient-matched marking guide and VSP for a vascularized fibula free flap reconstruction of a large sternal defect, highlighting how innovative solutions can improve surgical precision, clinical outcomes and the patient experience in complex reconstructive cases. To our knowledge, this is the first reported use of this technology for autologous chest wall reconstruction. However, further studies are needed to validate this approach and establish its role in chest wall reconstruction.

## Ethical approval

Ethical approval was obtained from the Metro South Health Research Ethics Committee with an exemption form, reference number EX/2025/QMS/122884.

## Patient consent

Written and verbal consent was obtained from the patient for this case report.

## Declaration of competing interest

The authors have no conflicts of interest to disclose.
